# LiDAR Point Cloud Recognition and Visualization with Deep Learning for Overhead Contact Inspection

**DOI:** 10.3390/s20216387

**Published:** 2020-11-09

**Authors:** Xiaohan Tu, Cheng Xu, Siping Liu, Shuai Lin, Lipei Chen, Guoqi Xie, Renfa Li

**Affiliations:** 1 Key Laboratory for Embedded and Network Computing of Hunan Province, Changsha 410082, China; txhan@hnu.edu.cn (X.T.); liusiping@hnu.edu.cn (S.L.); ls001@hnu.edu.cn (S.L.); lipeic@hnu.edu.cn (L.C.); xgqman@hnu.edu.cn (G.X.); lirenfa@hnu.edu.cn (R.L.); 2 College of Computer Science and Electronic Engineering, Hunan University, Changsha 410082, China

**Keywords:** convolutional neural networks (CNNs), deep learning, LiDAR (light detection and ranging), overhead contact components, point cloud recognition

## Abstract

As overhead contact (OC) is an essential part of power supply systems in high-speed railways, it is necessary to regularly inspect and repair abnormal OC components. Relative to manual inspection, applying LiDAR (light detection and ranging) to OC inspection can improve efficiency, accuracy, and safety, but it faces challenges to efficiently and effectively segment LiDAR point cloud data and identify catenary components. Recent deep learning-based recognition methods are rarely employed to recognize OC components, because they have high computational complexity, while their accuracy needs to be improved. To track these problems, we first propose a lightweight model, RobotNet, with depthwise and pointwise convolutions and an attention module to recognize the point cloud. Second, we optimize RobotNet to accelerate its recognition speed on embedded devices using an existing compilation tool. Third, we design software to facilitate the visualization of point cloud data. Our software can not only display a large amount of point cloud data, but also visualize the details of OC components. Extensive experiments demonstrate that RobotNet recognizes OC components more accurately and efficiently than others. The inference speed of the optimized RobotNet increases by an order of magnitude. RobotNet has lower computational complexity than other studies. The visualization results also show that our recognition method is effective.

## 1. Introduction

### 1.1. Background

**Figure 1:**
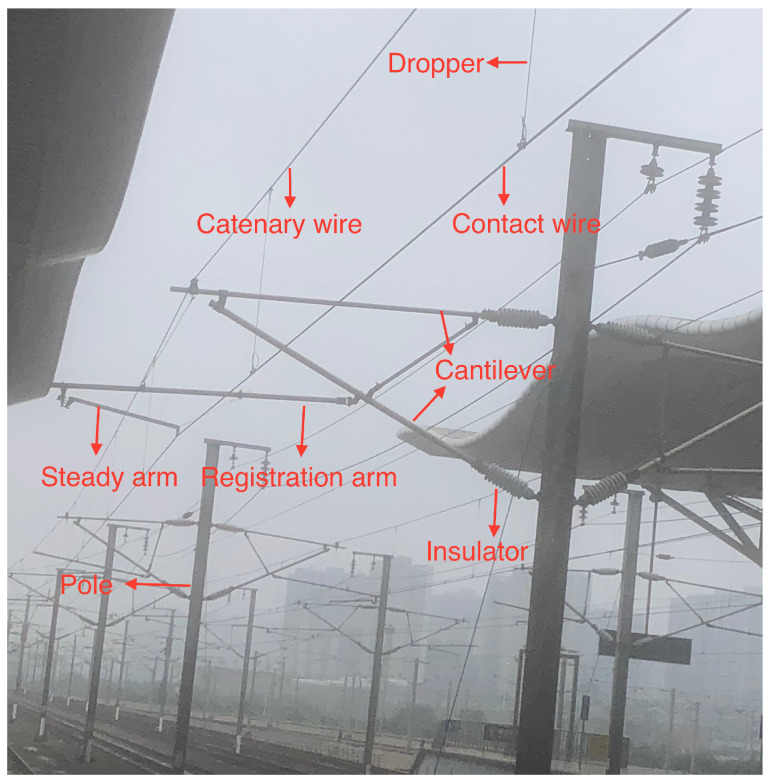
Overhead catenary components.

As one of the indispensable modes of transportation, high-speed railways have great significance to social and economic development. Recently, the scale of high-speed railways is expanding, and it is critical to ensure the safe and reliable operation of high-speed railways. As an important part of overhead catenary (OC) systems in high-speed railways, the catenary assumes the task of transmitting the electric energy from traction substations to trains. As shown in Figure 1, the OC system includes the contact wire, dropper, steady arm, registration arm, catenary wire, pole, cantilever, and insulator. The status of the catenary equipment is closely related to the normal operation of trains. Additionally, the faults and geometric parameters of the above components directly affect the safety of the train. However, the catenary is easily affected by natural and man-made factors, because it is exposed to the outdoor environment for a long time. The components in the OC systems may be broken and loose; their geometrical parameters may be abnormal and affect the normal operation of the train. Therefore, it is necessary to detect the catenary regularly, find out the existing faults, and repair them in time, ensuring that the catenary is safe.

Early catenary inspection was mainly carried out manually. This inspection method is slow in speed, big in measurement error, and low in efficiency. At the same time, it requires professional technicians to operate. To ensure the safety and reliability of high-speed railway operations, the efficiency of catenary inspection must be improved. Consequently, it is particularly urgent and essential to use intelligent inspection technology and automatic identification methods to detect contact lines. LiDAR (light detection and ranging) conducts non-contact measurement of the scene by continuously emitting laser beams, obtaining the distance information of the target point in the scene. Low-cost robots generally only use LiDAR to measure the catenary, such as the t-Cat hand-push device from TELICE in Spain and a series of catenary inspection products from Selectra Vision in Italy. The catenary inspection products usually first scan the fixed position of OC systems by LiDAR and obtain the point cloud. The OC systems are not blocked by unrelated wires or pillars, since railways strictly restrict the positions of all irrelevant objects. Second, from the point cloud of fixed OC systems, the geometric parameters of OC systems are calculated through coordinate conversion. Photogrammetry and LiDAR laser scanning are often combined for digital preservation of patrimonial and cultural heritage [1]. The combination also generates detailed visually aesthetic 3D models for historical cities [2]. For catenary inspection, only LiDAR is enough, because there is no need to display specific texture information [3] or generate realistic building models [4]. In general, the advantages of only using LiDAR are as follows:

(1) Low cost: LiDAR requires no auxiliary light sources and has low requirements on the working environment. LiDAR can be installed anywhere for catenary inspection. In contrast, vision measurement methods require more than two high-resolution cameras to generate stereo vision for catenary inspection. Additionally, cameras generally need to be supplemented by a light source and installed on detection trains for catenary inspection.

(2) High precision: LiDAR filters external interference with strong anti-interference performance and reliable measurement. LiDAR, such as the SICK LMS511-20100 used in this paper, can effectively filter insects, dust, and other small interfering objects. As summarized in the study [5], LiDAR is more accurate for measurement than photogrammetry.

Therefore, we focus on point cloud recognition instead of point cloud reconstruction [6]; for OC inspection, only LiDAR-based measurement reduces cost and improves precision relative to vision-based methods like [7]. However, the amount of point cloud data from LiDAR is large; it is a problem to efficiently and accurately identify catenary components from big data [8,9,10].

### 1.2. Motivation

Recently, there has been little research relying on the point cloud for overhead catenary (OC) inspection [11]. Researchers usually employ deep learning methods to process 2D (two-dimensional) images to predict the state of components in OC [12]. However, 2D image-based methods cannot measure the geometric parameters of OC, accurately. Relative to 2D image-based work, point cloud-based inspection can produce specific parameters for every component in OC, improving the accuracy of OC inspection [13]. Additionally, big point cloud data need to be processed, and catenary components need to be identified from these data to achieve automatic OC inspection. To process and recognize point cloud data, many studies use convolutional neural networks (CNNs) or fully connected layers. For example, Qi et al. [14] proposed PointNet based on fully connected layers, solving the disorder and irregularity of point cloud data. However, PointNet has limited generalization capabilities for complex scenes, due to the lack of extracting local features. To improve the effect of point cloud recognition, Qi et al. [15] designed PointNet++, and it recycles PointNet to extract multi-level local features. PointNet++ improves the lack of local feature extraction in PointNet, because it adopts the local area construction module. However, these deep learning methods are rarely used to recognize OC components for OC inspection. Besides, they still have the following shortcomings:

(1) The accuracy of these models for point cloud recognition should be improved, as we need to automatically identify multiple catenary components for complex OC systems. The model accuracy determines whether these complex OC components can be accurately identified.

(2) These models have high computational complexity, and they are hardly deployed on embedded devices such as intelligent inspection robots for automatic detection of OC systems. The embedded devices have limited computing resources; they not only require models to reduce computational complexity, but also demand models to meet delay constraints.

(3) Visualization software is still lacking for point clouds. As the amount of point cloud data is large in OC systems, it is difficult to display these point cloud data. The details of OC components cannot be automatically visualized.

The above restrictions prevent the development of OC component recognition on embedded devices [16,17,18] and the visualization of OC components. Additionally, embedded devices are resource constrained, and deep learning tasks consume multiple computing resources [19,20,21]. Recent work often leverages quantification or pruning methods to decrease the computational complexity of deep learning models. However, the quantification methods require integer-arithmetic-only devices; the pruning methods usually lose model accuracy. Therefore, a lightweight and high-precision model needs to be proposed [22,23], while model inference on devices should be accelerated without accuracy loss for faster point cloud recognition in OC systems.

### 1.3. Our Contribution

To solve the above challenges, we first propose a lightweight model, RobotNet, including 1D (one-dimensional) depthwise and pointwise convolutions and an attention module for OC component recognition, assisting in the automated inspection of OC systems. However, automated inspection of OC systems in high-speed railways is often carried out on embedded devices such as robots. To decrease the runtime of models on embedded devices, we then optimize RobotNet with the TVM [24] RPC (remote procedure call) tracker. The optimization respectively achieves 10.12× and 22.56× speedup on the TX2 GPU and the server GPU. Third, we design a software for the visualization of point cloud data. The visualization results qualitatively show that our RobotNet is more accurate than others. Extensive experiments demonstrate that our methods are precise, have low computational complexity, and are efficient. For example, our mean precision and intersection over union are respectively 0.16% and 0.38% higher than those of [13]. RobotNet respectively has at least 9.30% and 25.00% fewer parameters and MACs (multiply-and-accumulate operations)than others [13,14,15]. When the number of optimization trials is 1200, the optimized RobotNet has at least 99.42% less runtime than PointNet++ (MSG) and PointNet++ (SSG) [15].

We summarize the contribution of this paper as follows:

(1) We propose a lightweight model, RobotNet, which can be deployed on robots. RobotNet recognizes OC components in real high-speed railways. To decrease the computational complexity of RobotNet, we design the DPmodule, containing 1D depthwise and pointwise convolutions. The DP module not only extracts point cloud features, but also reduces the number of parameters and calculations. To increase the accuracy of RobotNet, we add the attention module to the feature extraction module in RobotNet. Different from other attention methods, our attention module includes no fully connected layers. It has few MACs and parameters, but can suppress unimportant features and strengthen important ones. Experiments demonstrate that our RobotNet is efficient, and it can accurately recognize OC components on embedded devices such as intelligent inspection robots.

(2) To accelerate inference on embedded devices, we optimize RobotNet with the TVM RPC tracker on the server, which improves optimization efficiency. Due to the powerful computing power of the server, the optimization is executed faster on the server than on embedded devices. After the optimization, the accuracy of RobotNet is unchanged, but its inference speed increases by an order of magnitude through the optimization. Additionally, the optimized RobotNet can be deployed multiple times on embedded devices and matches hardware efficiently. Experiments show that the optimized RobotNet spends two orders of magnitude less runtime than the state-of-the-art.

(3) We design software to facilitate the visualization of point cloud data. Our visualization software presents our point cloud recognition effect. This software can not only display a large amount of point cloud data, but also visualize the details of OC components. In this software, different colors are used to display the details of different three-dimensional (3D) components in OC systems. Additionally, we realize the functions to drag, zoom in, and zoom out the content in the software. Relying on the visualization, we compare the recognition results of different methods. The comparison results demonstrate that our recognition method is more accurate than others.

To the best of our knowledge, this is the first paper that provides an optimized model to recognize OC components. At the same time, we design a visualization software to display the details of catenary components.

## 2. Related Work

Research on point cloud recognition can be summarized into traditional and deep learning methods. The traditional methods are usually divided into clustering-based, model fitting, and region growing algorithms. For example, Zhou et al. [25] used Euclidean clustering and region-random sample consensus (RANSAC) to detect steady arms. Han et al. [26] also adopted RANSAC methods to segment cantilever structures and measured geometry parameters. These studies only recognize one component of OC. Guo et al. [27] designed a region growing method to segment point cloud data and classify the data with algorithms of the closest point search and KD tree for railway scenes. The above algorithms are restricted by prior knowledge, relying on handcrafted features. These methods are often leveraged for specified target inspection, as they have limitations to express statistical relations of point cloud data.

Compared with traditional methods, deep learning methods achieve promising results for point cloud processing and recognition. Due to the comparable effect of deep learning, researchers employed it to recognize the point cloud, aiming to overcome the challenges of the recognition. For example, Verdoja et al. [28] employed geometry and color-based supervoxels to segment the three-dimensional (3D) point cloud and understand scenes. Here, the supervoxels were obtained through point cloud conversion and acted as the inputs of deep learning models. Because the direct inputs of models are not the point cloud, the features of point cloud data are easily lost, and the calculation is increased. To reduce the calculation of models and make full use of the point cloud data information, some deep learning methods directly process and segment the point cloud [13,14,15].

Specifically, Lin et al. [13] relied on the k-nearest neighbor (kNN) algorithm to make full use of the local relationships among points for point cloud classification, but their model has high computational and model complexity. Qi et al. [14] designed PointNet with fully connected layers, segmenting irregular point cloud data. However, because PointNet ignores local structure information in adjacent point cloud points, it is restricted when generalizing to complex scenes. To track the problem of losing local feature extraction in PointNet, Qi et al. [15] proposed PointNet++, recycling PointNet to extract multi-level local features. As PointNet++ leverages a local area construction module, it overcomes the lack of local feature extraction in PointNet. These deep learning methods acquire comparable performance in point cloud segmentation, but they are rarely adopted to recognize complex OC components.

To inspect OC components, some works used 2D images of OC components as the inputs of deep learning and predicted the fault of components. Liu et al. [29] employed deep learning methods to detect the catenary support components and locate and find defects. This method relies on training with diverse images. Kang et al. [30] inspected the fault of insulator surfaces with deep CNN models, and the detection effect was comparable. However, these studies are based on high-resolution images. They take a long time to process the images. Additionally, their computational complexity is high, and they are difficult to adapt to the embedded devices for OC inspection. Compared with the methods mentioned above, this paper proposes a lightweight model to recognize the point cloud, optimizes the model to achieve inference acceleration, and provides a visualization software.

## 3. Proposed Methods

In Section 3.1, we design a lightweight model, RobotNet, based on the proposed attention module, processing the point cloud and recognizing OC components. In Section 3.2, to accelerate RobotNet, we optimize it with TVM on the intelligent inspection robot. In Section 3.3, we construct a visualization software to display OC components.

### 3.1. Point Cloud Recognition

**Figure 2:**
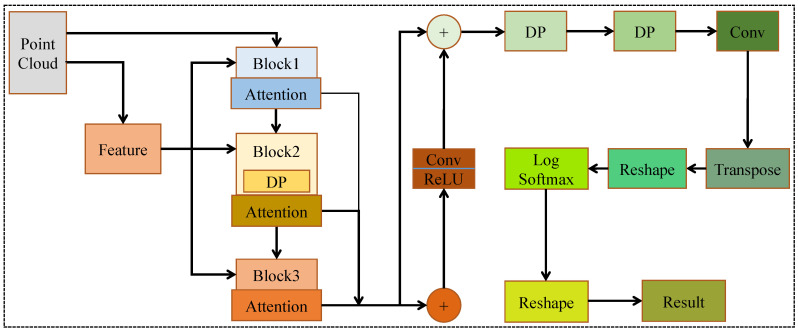
The proposed RobotNet model.

To process the point cloud and recognize OC components, we design the RobotNet model, as described in Figure 2. For point cloud data containing *n* points, we employ the feature model to extract their local features. The feature model is based on the kNN method in [13]. The dimension of each input point is three, because each input point consists of 3D coordinate values, namely x, y, and z. The feature model finds out the *k* neighboring points of each center point. Here, each input point is seen as a center point. Then, the feature model calculates the coordinate difference between adjacent points and the center point. The coordinate difference and point features are used as the input of Block1, Block2, and Block3. Block1 and Block3 both have three layers. Their first layer is a 2D convolution. Their second layer is the local feature module. The local feature module has a 1D convolution layer, which adopts the above coordinate difference as inputs and outputs a new coordinate difference. The new coordinate differences are multiplied by the features of *k* neighboring points, producing new features. These new features are summed. Then, the local feature module aggregates the summed features and the original features of the center point, generating the mixed features of the center point. The third layer of Block1 and Block3 is the 1D convolution. The third layer is responsible for extracting features again for the center point.

Different from Block1 and Block3, the third layer of Block2 is a DP module. We design the DP module, inspired by depthwise separable convolution in image processing. The DP module consists of the depthwise and pointwise layers. These layers contain 1D convolutions with different types of kernels. The size and depth of the kernels change. In detail, the size of the kernels is respectively three and five in the depthwise and pointwise layers; the depth of the kernels is respectively 128 and 64 in the depthwise and pointwise layers. The different size and depth of kernels show that different scales of detailed features can be extracted by DP. Additionally, DP reduces the number of parameters and calculations through group convolution in depthwise layers. The first and second layers of Block2 are the same as those of Block1 and Block3. To increase the nonlinear relationship in Block1, Block2, and Block3, each 1D or 2D convolution is followed by a ReLU layer.

**Figure 3:**
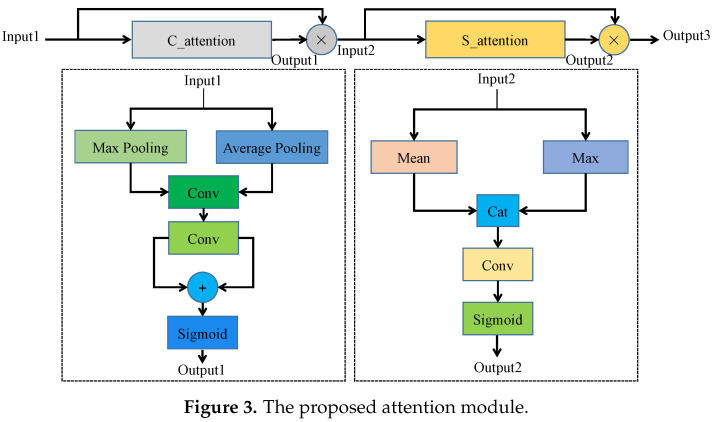
The proposed attention module.

After Block1, Block2, and Block3, there is an attention module. We propose the attention module inspired by Woo et al. [31], improving accuracy in image detection and classification tasks. The structure of the proposed attention is shown in Figure 3. The attention module learns important features from each block module and suppresses unnecessary ones. The attention module consists of two parts. The first part C_attention focuses on channel attention; the second part focuses on spatial attention. The output of C_attention is used as the input of the second part S_attention. The convolutions of the two parts are all 1D. In C_attention, the kernel size of 1D convolutions is one. In the second part S_attention, the kernel size of 1D convolutions is seven. The size of the inputs is unchanged with the attention module. The attention module effectively helps information flow within RobotNet.

Specifically, in C_attention, we use a max and average pooling. They respectively generate max-pooled Output1 and average-pooled features Output2, as illustrated in Figure 3. The 1D channel attention feature is produced as follows:

(1) $$\mathrm{Output}1=\delta \left(C_{1}\left(C_{0}\left(P_{\mathrm{avg}}\right)\right)+C_{1}\left(C_{0}\left(P_{\max}\right)\right)\right),$$

where $P_{\mathrm{avg}}$ is the average pooling layer; $P_{\max}$ is the max pooling layer. The outputs from the two layers are both employed as the inputs of 1D convolution $C_{0}$. To reduce the number of MACs and parameters, we set the output channels of $C_{0}$ to $\frac{1}{16}$ of the input channels; $C_{1}$ denotes another 1D convolution; δ represents the sigmoid function. The 1D spatial attention feature is produced as follows:

(2) $$\mathrm{Output}2=\delta \left(C\left(\left[P_{\max};P_{\mathrm{mean}}\right]\right)\right),$$

where [] denotes the concatenation operation; $P_{\mathrm{mean}}$ and $P_{\max}$ are respectively the mean and max pooling layers; C represents the 1D convolution, which has a 7 × 7 kernel.

To enhance useful features and suppress unimportant features, we respectively multiply the spatial and channel attention features by the inputs Input1 and Input2:

(3) Input2=Output1×Input1;

(4) Output3=Output2×Input2.

The multiplication operation makes unimportant features have low weights and critical features have high weights. Therefore, the attention module enhances the representational power of RobotNet. Additionally, the attention module has little calculation. For example, the C_attention and S_attention modules respectively have 512 and 14 parameters; the C_attention and S_attention modules both have about zero MACs.

After the three attention modules, we obtain three types of features. These features are fused and used as the inputs of a 1D convolution layer for the extraction of global features. Then, we design a jump connection structure, as depicted in Figure 2. This structure can combine point features of different layers, making the extracted point cloud features and global features more abundant. To further learn different scales of detailed features with less calculation, we employ two DP modules. To reduce the dimensions of features, we adopt a 1D convolution layer. To adjust the size of features, we perform a transpose and reshape operation. At the end of RobotNet, we use a log softmax and reshape operation, outputting the category of each point cloud.

### 3.2. Optimizing Model

**Figure 4:**
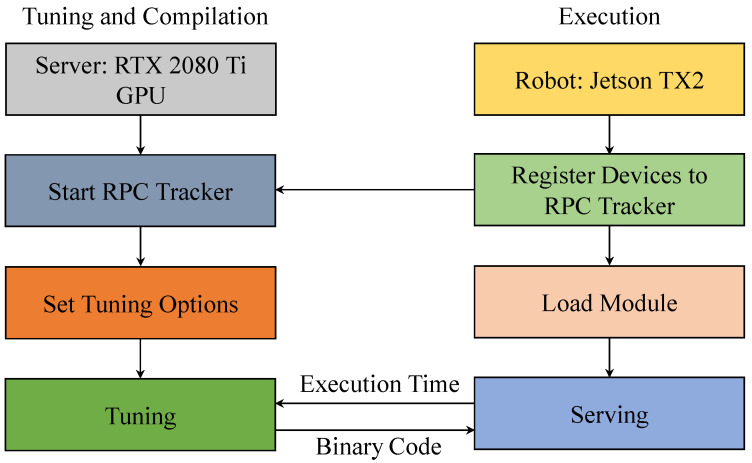
TVM tuning through the RPC (remote procedure call) tracker.

In this section, we optimize RobotNet with TVM [24]. To accelerate the optimization, we perform TVM tuning through the RPC (remote procedure call) tracker on the server GPU, as depicted in Figure 4. On the server, including a RTX 2080 Ti GPU, we start the RPC session. Then, the robot including Jetson TX2 is registered to the server. On the robot, some options are set for tuning. The options contain the number of trials, overtime time, device key, port number, IP address of the server, file name of the log file, and tuning algorithm. Here, when the number of trials is larger, the optimizing process will be executed for a long time. After the configuration is finished, tuning tasks are extracted from RobotNet for tuning. When tuning, we create a tuner on the server. The server generates binary code and conveys it to the robot. The robot evaluates the execution time of RobotNet, sending the execution time to the server. The server continues tuning, until it completes the trials. Then, the robot completes serving. The execution time on the robot is the shortest when RobotNet best matches the robot.

**Figure 5:**
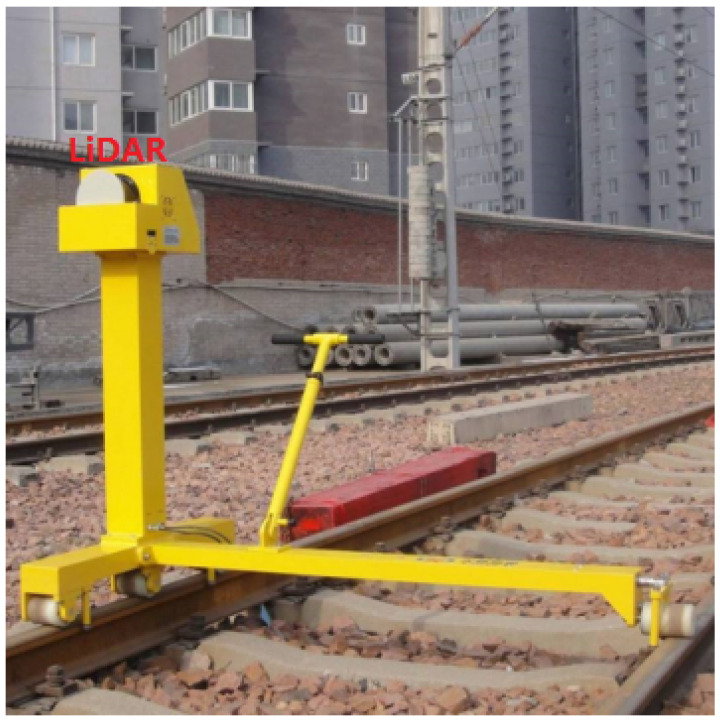
Intelligent inspection robots.

The RPC tracker accelerates optimization. There are two reasons. First, the server has a strong CPU and GPU for faster tuning than robots. With the help of the server’s computing power, tuning tasks are launched and executed more rapidly on the server than on the robot. Second, various devices can be connected to the server for tuning at the same time. The tuning in parallel accelerates the optimization, compared with single tuning. After the optimization is complete, the inference time of RobotNet is greatly reduced, as compared with that before optimization. Although the inference time decreases, the accuracy of RobotNet is unchanged. The number of MACs and parameters is also unchanged. The tuned RobotNet will be deployed multiple times on numerous robots, when the overhead catenary needs to be inspected by the robots, as depicted in Figure 5. Therefore, the server sacrifices one-time computing resource consumption, but it brings multiple deployments and rapid inference of RobotNet to the robots.

### 3.3. Constructing Visualization Software for Point Cloud Recognition

To facilitate the visualization of point cloud data, we construct the software. This is also conducive to qualitatively show the effect of our recognition methods and recognize OC components. In general, the overhead catenary is long; the railway scene is complex; and the catenary components are numerous. Then, the number of point cloud data is very large. For example, the number of point cloud data can reach 100,000 for several hundred meters of railway. If we visualize the data point by point, the workload is very large. To efficiently visualize point cloud data, we construct the software.

**Figure 6:**
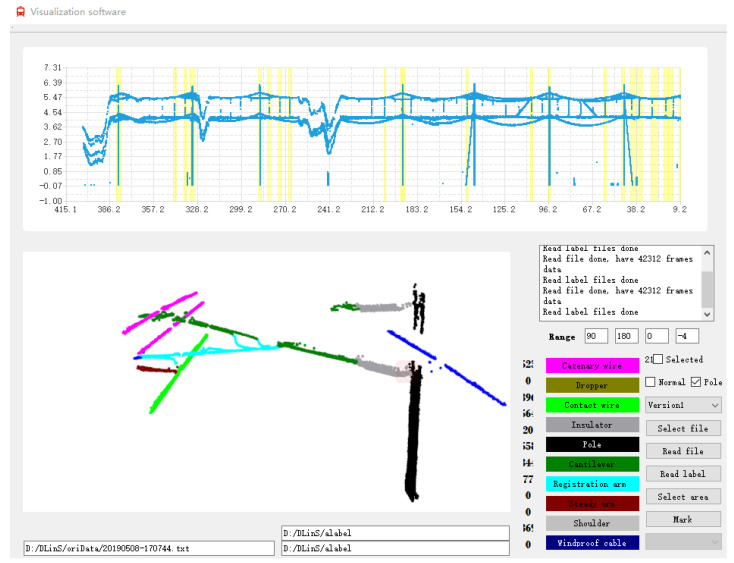
Constructing visualization software for point cloud recognition.

This visualization software can easily display the effect of point cloud recognition. Through visualization, the internal structure of the overhead catenary is recognized. As shown in Figure 6, the upper part of this figure is a two-dimensional (2D) view of the point cloud data about the overhead contact. Here, the number on the vertical axis represents the value of the point cloud on the Y-axis; the number on the horizontal axis represents the value of the point cloud on the Z-axis. For the value of the point cloud on the X-, Y-, and Z-axis, the unit is meter (m). The 2D view reflects the projection of point cloud data on the yz plane.

The lower part of Figure 6 is a 3D view of the point cloud data. In the 3D view, the X-axis is along the horizontal axis, from left to right; the Y-axis is straight up; the Z-axis is perpendicular to the X-axis and Y-axis, denoting the moving direction of the intelligent inspection robot. We use different colors to represent different 3D components in OC systems. For example, the red point cloud data represent the catenary wire, black point cloud data the pole, and gray point cloud data the insulator.

The visualization algorithm is shown in Algorithm 1. The programming tool is Qt Creator; the programming language is C++. We obtain the 3D coordinate of the 3D point cloud data from Steps 1–6 of this algorithm. In detail, suppose that $P_{i}$ is the *i*th frame of 2D LiDAR data:

(5) $$P_{i}=\left\{\left(r_{i,j},a_{i,j}\right)|j=1,\ldots ,n\right\},$$

where $P_{i}$ is the *i*th frame of 2D LiDAR data. One frame of 2D LiDAR data is composed of measured distance values at multiple angles $a_{i,j}$ (j=1,…,n). Here, *n* is the number of angle values; $a_{i,j}$ is an angle value in the *i*th frame; $r_{i,j}$ is the measured distance in the angular $a_{i,j}$.
**Algorithm 1** Visualization algorithm**Input:** files that store point cloud data**Output:** visualization results1:Set the control button “select file” to realize the function of selecting the file path2:Set the control button “read file” to realize the function of reading the file from the above file path3:**if** the point cloud data are the raw data collected by the 2D SICK LiDAR sensor **then**4:    convert the 2D point cloud data into 3D data5:    obtain the 3D coordinate of the 3D data6:**end if**7:Process each row of 3D data8:Store all frames in the list *A*, and obtain *Z* frames of data9:Set the scale range of the X-axis and Y-axis, and display the coordinate axes10:**for***z* = 1, *Z* **do**11:    take out the coordinates of each frame of data12:    assign the above coordinates to the display control $D_{1}$13:**end for**14:Achieve the function of the display control $D_{2}$15:Mark the display content of $D_{2}$ with different colors to display the different OC components16:Realize the functions of dragging, zooming in, and zooming out for the display controls $D_{1}$ and $D_{2}$

Suppose that $Q_{i}$ is the *i*th frame of 3D point cloud data converted from the *i*th frame of 2D LiDAR data:

(6) $$Q_{i}=\left\{\left(x_{i,j},y_{i,j},z_{i,j}\right)|j=1,\ldots ,n\right\},$$

where $x_{i,j}$, $y_{i,j}$, and $z_{i,j}$ are respectively *x*, *y*, and *z* coordinates.

In the process of converting 2D data $P_{i}$ to 3D data $Q_{i}$, we adopt the following calculation method:

(7) $$\left[\begin{array}{c} x_{i,j}\\ y_{i,j}\\ z_{i,j} \end{array}\right]=r_{i,j}\left[\begin{array}{c} -\cos(a_{i,j})\\ \sin(a_{i,j})\\ 0 \end{array}\right]+\left[\begin{array}{c} 0\\ 0\\ d_{i} \end{array}\right],$$

where $d_{i}$ is the moving distance of the intelligent inspection robot in the *i*th frame. Then, we acquire the 3D coordinate of the 3D point cloud data from 2D data. In Step 7 of Algorithm 1, the same *z*-value data are divided into one frame, and different *z*-value data are divided into different frames. From Steps 8–13 of this algorithm, we display the point cloud data in the upper part of Figure 6. In Step 14, we achieve the function of $D_{2}$ to select the display content of the display control $D_{1}$ and show the details of the content. For the display content of $D_{2}$, different overhead catenary components are marked with different colors, as depicted in the lower part of Figure 6. After that, we realize the functions to drag, zoom in, and zoom out the display content of the controls $D_{1}$ and $D_{2}$.

## 4. Experiments

In this section, we describe the experimental details, with the experimental environment in Section 4.1, the datasets in Section 4.2, and the evaluation metrics in Section 4.3.

### 4.1. Experimental Environment

**Figure 7:**
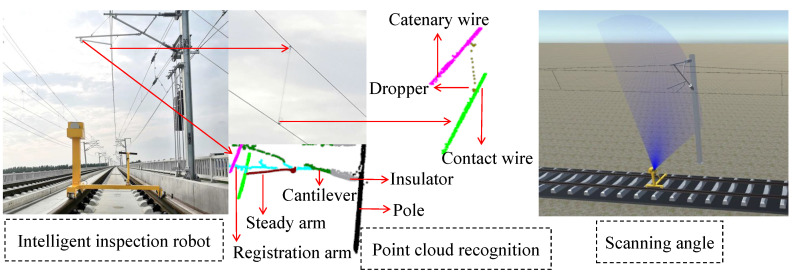
LiDAR scanning angle and point cloud recognition.

The models in this paper are trained on the server. The server includes Intel^®^ Core^™^ i9-9900K CPU, Intel Z390 motherboard, and RTX 2080 Ti GPU. The frame buffer of the GPU is 11GB GDDR6. The model inference is performed on the intelligent inspection robot, as shown in Figure 5. The robot is equipped with a 2D SICK LiDAR (LMS511-20100) and a Jetson TX2. The Jetson TX2 has an NVIDIA Pascal^™^ GPU, and the number of CUDA cores is 256. The CPUs of the Jetson TX2 are dual-Core NVIDIA Denver and quad-Core ARM. The Jetson TX2 is responsible for model inference. In the process of model training and inference, the Max-N mode on TX2 is set. The LiDAR is responsible for measuring the overhead catenary. For model training and inference, the PyTorch (https://pytorch.org/) framework is employed. The loss function is NLLLoss in PyTorch. For training, when the overall loss from the loss function becomes stable, the model training reaches an ideal state. Through empirical experiments, we found that our model training reaches the ideal state when the epoch is 251. We also display the scanning angle of LiDAR (90 degrees) and the process of point cloud recognition in Figure 7.

### 4.2. Datasets

To train and test the proposed model, we use the datasets collected and preprocessed by Lin et al. [13]. The dataset is collected by a 2D LiDAR (SICK LMS511-20100), which is installed on an intelligent inspection robot. The scanning angle of the LiDAR is 90 degrees, which can filter out some irrelevant objects. The scanning plane of the LiDAR sensor is perpendicular to the rail direction, facing the catenary and measuring the catenary. The LiDAR sensor generates point cloud data. Each frame of the point cloud data is converted into 3D data for data annotation and model training. In the 3D plane, the XY direction is the same as the scanning direction of the LiDAR sensor. The Z direction is the moving direction of the intelligent inspection robot. Lin et al. [13] manually labeled the 3D data and removed noise. Balsa-Barreiro and Lerma [32] mentioned that the distribution pattern and point density have an impact on point cloud data. Lin et al. [13] summed and averaged the 3D coordinate value of previous and next frames. This method helps overcome the above impact. As the 2D SICK LiDAR scans 25 frames per second, the speed is fast. There is little difference between the 3D coordinate values of the previous and next frame, and the methods of summing and averaging can reduce the impact of different point density.

In the dataset [13], the distance of labeled data is 16 km. The labeled data are split into a training set and a testing set. Different from vision-based methods reducing noise [3,33], the data [13] used in this paper are directly denoised by the LiDAR or algorithms. Specifically, the LiDAR (SICK LMS511-20100) used to produce data can effectively filter insects, dust, and other small interfering objects. For outliers, hashed, and other abnormal points in the original data, they are filtered with the PassThrough algorithm in PCL (https://pointclouds.org/documentation) (Point Cloud Library). For unrelated wires or pillars that cannot be filtered by the LiDAR or algorithms, they are labeled as a noise category. The noise category does not affect the robot inspecting the catenary components, because robots do not scan the noise category. In detail, the railway restricts the position of the catenary, and the position of catenary components is fixed relative to the railway tracks. On the contrary, the position of the noise category is not fixed. The robot only pays attention to the LiDAR data at a fixed position relative to the railway tracks, so Lin et al. [13] did not classify the noise category, specifically. They only identified catenary components, which are not blocked by unrelated wires or pillars because of the strict environment of railways. Following Lin et al. [13], we also did not classify the noise category, specifically.

### 4.3. Metrics

Based on the existing evaluation methods of point cloud recognition with deep learning, we use the metrics of precision, intersection over union (IoU), and mean accuracy (MA) to evaluate the accuracy:

- $precision=\frac{TP}{TP+FP}$,
- $\mathrm{IoU}=\frac{TP}{TP+FP+FN}$,

where FP, FN, and TP respectively represent false positive, false negative, and true positive counts. In addition, we adopt the common metric of mean accuracy (MA), which is the average of the overall accuracy of all categories. We also evaluate the inference runtime and the number of parameters/MACs of all methods.

These metrics are widely employed in semantic segmentation, image classification, and object detection. For the runtime, parameters, and MACs, lower is better. For precision, IoU, and MA, higher is better.

## 5. Results and Analysis

In this section, we assess our methods. We compare the recognition results of different methods in Section 5.1. We evaluate the number of MACs and parameters in Section 5.2. We compare the inference runtime of different models on two platforms in Section 5.3. We explore the effect of the number of optimizing trials on model runtime on the TX2 GPU in Section 5.4. We visualize the point cloud recognition results of different models by using our visualization software in Section 5.5.

### 5.1. Comparison of the Point Cloud Recognition Results

As shown in Table 1, we compared the recognition precision (precision) and intersection over union (IoU) of different methods on the test dataset. Bold values in this table represent the best recognition results in each category. Following Lin et al. [13], we set the number of neighboring points in the kNN method to 16. For the dropper and insulator, PointNet++ performed better than our methods. The reason is that the precision and IoU are higher when the number of neighboring points in the local area is greater. The number of neighboring points in the local area was set to 32, while ours was 16. The precision and IoU of our various components were better than [14]. For most components, our performance in precision and IoU outperformed other methods. In detail, our precision of the contact wire, steady arm, catenary wire, pole, and cantilever was higher than that of the state-of-the-art [13,14,15]. As another example, our precision and IoU were respectively 0.10% and 0.15% higher than those of [13] for the contact wire. Therefore, our method is more practical than the state-of-the-art in precision and IoU for the OC system.

### 5.2. Comparison of the Number of MACs and Parameters

**Figure 8:**
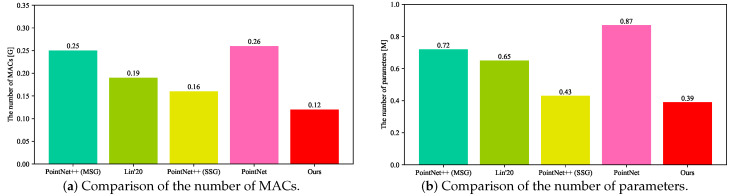
Comparison of the number of MACs (multiply-and-accumulate operations) and parameters.

Comparison of the Number of MACs. We evaluated the number of MACs as shown in Figure 8a. The ‘[G]’ in this figure denotes $10^{\wedge }9$. The number of MACs reflects the computational complexity of the models. Compared with PointNet++ (SSG) [15], the MACs of our model were reduced at least 25.00%. This result shows that our model has less computational complexity and takes up less computational overhead. Despite with fewer MACs, our mean recognition precision and intersection over union were still better than the other methods. Here, the inputs of all models were all 300 point cloud points. The analysis and results in Figure 8a demonstrate that our computational complexity was the lowest among those of the state-of-the-art.

Comparison of the Number of Parameters. We evaluated the number of parameters as shown in Figure 8b. The ‘[M]’ in this figure denotes $10^{\wedge }6$. The number of parameters reflects the model complexity. The results in Figure 8b show that our model had the lowest model complexity among all comparison methods, because we had at least 9.30% fewer parameters than the others [13,14,15]. The fewer parameters show that the model occupies less memory space and storage space. Here, the inputs of all models were all 300 point cloud points. The input has no relationship with the number of parameters. Despite the fewer parameters, our mean recognition precision and intersection over union were still better than the other models.

Our fewer parameters and MACs benefit from the lightweight design, which is specifically implemented through the DP module. At the same time, we improved the accuracy through the proposed attention module. This attention module suppresses unimportant information and strengthens important information. Then, while keeping the computational and model complexity unchanged, we optimized the model with TVM. The optimization not only keeps the accuracy constant, but also speeds up the inference speed of our model. We will demonstrate our inference speed before and after optimization in the following section.

### 5.3. Comparison of Inference Runtime on Different Platforms

**Figure 9:**
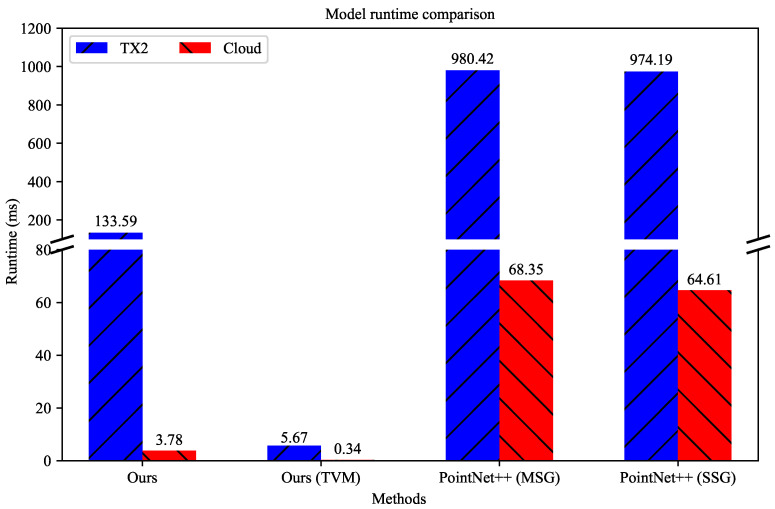
Runtime comparison.

On the TX2 GPU and server GPU, we respectively assessed the inference runtime of RobotNet and compared our results with those of other methods. The comparison is depicted in Figure 9. In this figure, “Ours” represents RobotNet without optimization; “Ours (TVM)” denotes our optimized RobotNet; the number of optimizing trials was 1200. The Y-axis has a portion cut out. The broken Y-axis can display the results of different orders of magnitude. The inference runtime on the TX2 GPU and server GPU had different orders of magnitude, so we adopted the broken Y-axis to display the inference runtime. For comparison, we implemented PointNet++ (MSG) and PointNet++ (SSG) and measured their inference runtime. The input of all methods was 1000 point cloud points on the TX2 GPU and server GPU.

As shown in Figure 9, our models are lightweight. The RobotNet before and after optimization both outperforms the state-of-the-art in runtime whether on the server GPU or TX2 GPU. On the TX2 GPU, the runtime of our RobotNet is lower than that of other methods. In detail, RobotNet has an 86.37% and an 86.29% lower runtime than PointNet++ (MSG) and PointNet++ (SSG). Optimized by TVM, RobotNet reduces the runtime by 22.56 times. On the server GPU, the runtime of our RobotNet is also better than that of the other methods. Specifically, RobotNet has at least 94.15% less runtime than PointNet++ (MSG) and PointNet++ (SSG). The optimized RobotNet obtains a 10.12× speedup compared with that before optimization. Therefore, our optimized RobotNet is more efficient than the state-of-the-art.

### 5.4. Effect of the Number of Optimizing Trials on Runtime

**Figure 10:**
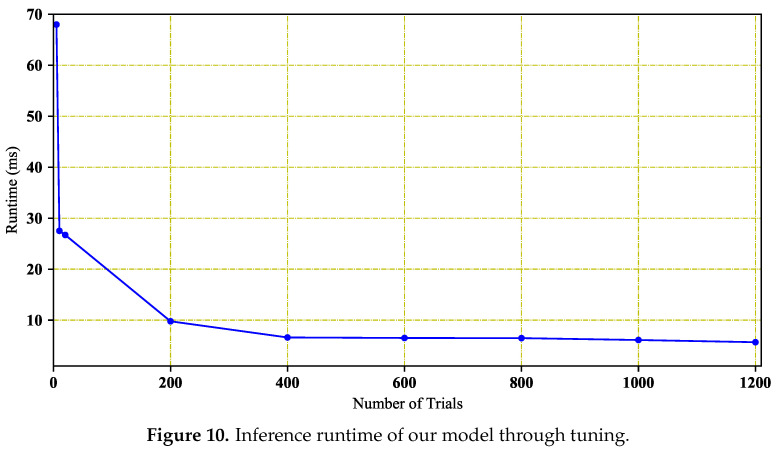
Inference runtime of our model through tuning.

In this section, we explore the impact of the number of optimizing trials on the runtime. Here, the experiment platform was the TX2 board on the intelligent inspection robot. On the TX2, we used its GPU to perform optimization and inference. The input of our methods before and after optimization was 1000 point cloud points. TVM proved that the tuner based on machine learning searches suitable configurations more rapidly than random search, so we employed a machine learning algorithm, XGBoost, to find a better match with the TX2 GPU. As shown in Figure 10, the results of the number of optimizing trials are listed. This figure shows that when the number of optimizing trials is less than 400, the runtime of RobotNet decreases rapidly; when the number of optimizing trials is more than 400, the runtime of RobotNet tends to stabilize. When the number of optimizing trials is 1200, the runtime is the lowest on the TX2 GPU. Here, the inference runtime is lower, when RobotNet matches the hardware better. Therefore, when the number of optimizing trials is 1200, the optimized RobotNet is best suited for the TX2 GPU.

### 5.5. Comparison of the Visualized Results

**Figure 11:**
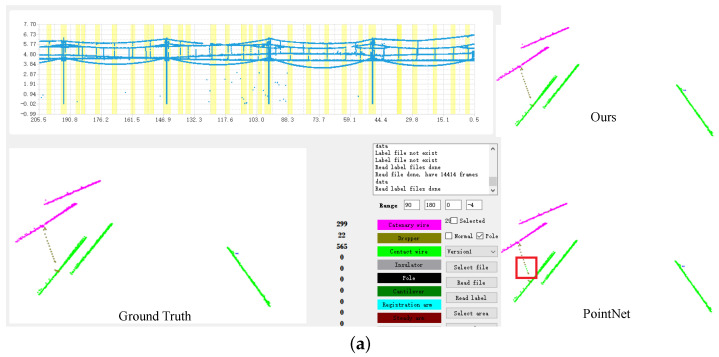

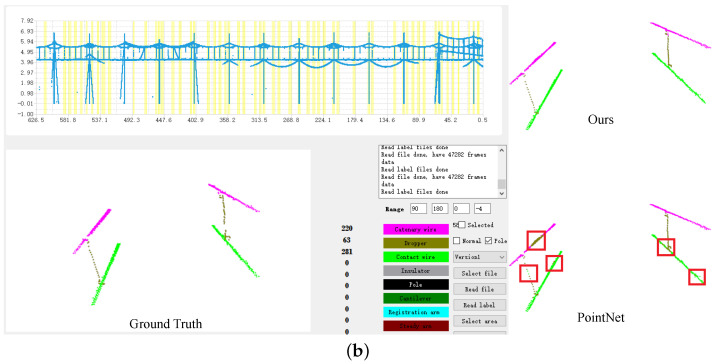
(**a**) Comparison of the visualized results; (**b**) Comparison of the visualized results.

The recognition results of RobotNet are shown in Figure 11a,b. The visualization results in our software can clearly present the recognition results. In the lower left corner of the visualization software, the details of the recognition result are displayed; different components are represented by different colors. The recognition results of different models were obtained by performing model inference on the test dataset. We also implemented the methods of Qi et al. [14] and visualized their results. We use boxes to mark the difference between PointNet and our RobotNet. The qualitative comparison shows that our RobotNet performs better than PointNet. For example, RobotNet recognizes the dropper, contact wire, and catenary wire more accurately than PointNet; while PointNet recognizes a dropper as a contact wire, as shown in Figure 11a. As another example, PointNet recognizes a catenary wire as a dropper, as in Figure 11b. In addition, the recognition result of RobotNet is clear, close to the ground truth. Based on our visualization software, we can easily distinguish different components in real OC systems. The above comparison of the visualization results confirms that the recognition accuracy of RobotNet is high.

## 6. Conclusions

For overhead contact inspection, we propose a lightweight model, RobotNet, to process point cloud data from LiDAR and recognize OC components. Our RobotNet relies on depthwise and pointwise convolutions and an attention module. The depthwise and pointwise convolutions not only extract point cloud features, but also reduce the computational complexity of the models. The attention module improves recognition accuracy by suppressing unimportant features and strengthening important ones. For fast point cloud recognition on embedded devices, we optimize RobotNet with the TVM RPC tracker on the server. The RPC improves optimization efficiency. With the help of the powerful computing power of the server GPU, we complete the optimization faster on the server than on the TX2 GPU. For the visualization of point cloud data, we design the software. This software displays a large amount of point cloud data, while visualizing the details of the OC components. Additionally, the software can drag, zoom in, and zoom out its display content. Experiments demonstrate that our methods are efficient and effective. RobotNet has less computational complexity than other studies. The optimized RobotNet spends two orders of magnitude less runtime than the state-of-the-art. The visualization results of RobotNet are also more accurate than other methods. Our main purpose is to design algorithms for LiDAR point cloud recognition here, so we did not focus on LiDAR point density and digital elevation models. Effective LiDAR scanning systems should be designed to reduce the impact of distribution pattern and point density on digital elevation models; to achieve this, we will follow Balsa-Barreiro and Lerma [32].

## Figures and Tables

**Table 1:** Comparison of the point cloud recognition results.

Point Cloud Category	Ours		PointNet++ (SSG)		Lin et al. [13]		PointNet++ (MSG)		PointNet	
	Precision	IoU	Precision	IoU	Precision	IoU	Precision	IoU	Precision	IoU
Contact wire	**99.89**	**99.75**	99.37	98.28	99.79	99.60	99.51	98.47	99.14	98.74
Dropper	97.16	93.68	**97.22**	93.15	96.39	91.91	96.96	**93.72**	89.67	76.84
Steady arm	**95.97**	85.68	91.12	83.33	95.27	**86.44**	90.80	84.51	92.02	82.23
Registration arm	94.70	91.38	**95.42**	89.83	95.23	**92.11**	95.39	90.61	93.59	88.64
Catenary wire	**99.65**	**99.51**	98.57	97.93	99.45	99.16	98.61	98.06	98.99	98.12
Pole	**99.91**	**99.76**	99.85	99.65	99.80	99.64	99.86	99.64	99.73	99.34
Cantilever	**93.87**	**88.68**	92.60	87.88	92.81	87.23	93.38	87.75	87.03	77.87
Insulator	97.18	94.12	97.26	**94.45**	**97.36**	93.59	97.10	94.21	96.03	91.68
Mean accuracy	**97.17**	**94.07**	96.42	93.06	97.01	93.71	96.45	93.37	94.52	89.18

## References

[1] Owda A., Balsa-Barreiro J., Fritsch D. (2018). Methodology for digital preservation of the cultural and patrimonial heritage: Generation of a 3D model of the Church St. Peter and Paul (Calw, Germany) by using laser scanning and digital photogrammetry. Sens. Rev..

[2] Balsa-Barreiro J., Fritsch D. (2018). Generation of visually aesthetic and detailed 3D models of historical cities by using laser scanning and digital photogrammetry. Digit. Appl. Archaeol. Cult. Herit..

[3] Tang Y., Chen M., Lin Y., Huang X., Huang K., He Y., Li L. (2020). Vision-Based Three-Dimensional Reconstruction and Monitoring of Large-Scale Steel Tubular Structures. Adv. Civ. Eng..

[4] Balsa-Barreiro J., Fritsch D. (2015). Generation of 3D/4D Photorealistic Building Models. The Testbed Area for 4D Cultural Heritage World Project: The Historical Center of Calw (Germany). International Symposium on Visual Computing (ISVC).

[5] Mueller A.R. LiDAR and Image Point Cloud Comparison. https://calhoun.nps.edu/handle/10945/43960.

[6] Tang Y., Li L., Wang C., Chen M., Feng W., Zou X., Huang K. (2019). Real-time detection of surface deformation and strain in recycled aggregate concrete-filled steel tubular columns via four-ocular vision. Robot. Comput. Integr. Manuf..

[7] Chen M., Tang Y., Zou X., Huang K., Li L., He Y. (2019). High-accuracy multi-camera reconstruction enhanced by adaptive point cloud correction algorithm. Opt. Lasers Eng..

[8] Duan M., Li K., Liao X., Li K. (2018). A Parallel Multiclassification Algorithm for Big Data Using an Extreme Learning Machine. IEEE Trans. Neural Netw. Learn. Syst..

[9] Chen J., Li K., Tang Z., Bilal K., Yu S., Weng C., Li K. (2017). A Parallel Random Forest Algorithm for Big Data in a Spark Cloud Computing Environment. IEEE Trans. Parallel Distrib. Syst..

[10] Chen C., Li K., Ouyang A., Li K. (2018). FlinkCL: An OpenCL-Based In-Memory Computing Architecture on Heterogeneous CPU-GPU Clusters for Big Data. IEEE Trans. Comput..

[11] Chen L., Xu C., Lin S., Li S., Tu X. (2020). A Deep Learning-Based Method for Overhead Contact System Component Recognition Using Mobile 2D LiDAR. Sensors.

[12] Kang G., Gao S., Yu L., Zhang D., Wei X., Zhan D. (2019). Contact Wire Support Defect Detection Using Deep Bayesian Segmentation Neural Networks and Prior Geometric Knowledge. IEEE Access.

[13] Lin S., Xu C., Chen L., Li S., Tu X. (2020). LiDAR Point Cloud Recognition of Overhead Catenary System with Deep Learning. Sensors.

[14] Qi C.R., Su H., Mo K., Guibas L.J. PointNet: Deep Learning on Point Sets for 3D Classification and Segmentation. Proceedings of the IEEE Conference on Computer Vision and Pattern Recognition (CVPR).

[15] Qi C.R., Yi L., Su H., Guibas L.J. PointNet++: Deep Hierarchical Feature Learning on Point Sets in a Metric Space. Proceedings of the 31st International Conference on Neural Information Processing Systems.

[16] Xie G., Zeng G., Jiang J., Fan C., Li R., Li K. (2020). Energy management for multiple real-time workflows on cyber—Physical cloud systems. Future Gener. Comput. Syst..

[17] Tu X., Xu C., Liu S., Li R., Xie G., Huang J., Yang L. (2020). Efficient monocular depth estimation for edge devices in internet of things. IEEE Trans. Ind. Informat..

[18] Chen W., Xie G., Li R., Bai Y., Fan C., Li K. (2017). Efficient task scheduling for budget constrained parallel applications on heterogeneous cloud computing systems. Future Gener. Comput. Syst..

[19] Chen W., An J., Li R., Fu L., Xie G., Bhuiyan M.Z.A., Li K. (2018). A novel fuzzy deep-learning approach to traffic flow prediction with uncertain spatial—Temporal data features. Future Gener. Comput. Syst..

[20] Nai K., Xiao D., Li Z., Jiang S., Gu Y. (2019). Multi-pattern correlation tracking. Knowl. Based Syst..

[21] Li G., Peng M., Nai K., Li Z., Li K. (2020). Reliable correlation tracking via dual-memory selection model. Inf. Sci..

[22] Xie G., Zeng G., Xiao X., Li R., Li K. (2017). Energy-Efficient Scheduling Algorithms for Real-Time Parallel Applications on Heterogeneous Distributed Embedded Systems. IEEE Trans. Parallel Distrib. Syst..

[23] Huang J., Li R., An J., Ntalasha D., Yang F., Li K. (2017). Energy-Efficient Resource Utilization for Heterogeneous Embedded Computing Systems. IEEE Trans. Comput..

[24] Chen T., Moreau T., Jiang Z., Zheng L., Yan E., Cowan M., Shen H., Wang L., Hu Y., Ceze L. TVM: An Automated End-to-End Optimizing Compiler for Deep Learning. Proceedings of the 13th USENIX Conference on Operating Systems Design and Implementation.

[25] Zhou J., Han Z., Wang L. A Steady Arm Slope Detection Method Based on 3D Point Cloud Segmentation. Proceedings of the IEEE 3rd International Conference on Image, Vision and Computing (ICIVC).

[26] Han Z., Yang C., Liu Z. (2019). Cantilever Structure Segmentation and Parameters Detection Based on Concavity and Convexity of 3D Point Clouds. IEEE Trans. Instrum. Meas..

[27] Guo B., Yu Z., Zhang N., Zhu L., Gao C. (2017). 3D point cloud segmentation, classification and recognition algorithm of railway scene. Chin. J. Sci. Instrum..

[28] Verdoja F., Thomas D., Sugimoto A. Fast 3D point cloud segmentation using supervoxels with geometry and color for 3D scene understanding. Proceedings of the IEEE International Conference on Multimedia and Expo (ICME).

[29] Liu Z., Zhong J., Lyu Y., Liu K., Han Y., Wang L., Liu W. Location and fault detection of catenary support components based on deep learning. Proceedings of the IEEE International Instrumentation and Measurement Technology Conference (I2MTC).

[30] Kang G., Gao S., Yu L., Zhang D. (2019). Deep Architecture for High-Speed Railway Insulator Surface Defect Detection: Denoising Autoencoder With Multitask Learning. IEEE Trans. Instrum. Meas..

[31] Woo S., Park J., Lee J.Y., Kweon I.S. CBAM: Convolutional Block Attention Module. Proceedings of the European Conference on Computer Vision.

[32] Balsa-Barreiro J., Lerma J.L. (2014). Empirical study of variation in lidar point density over different land covers. Int. J. Remote Sens..

[33] Tang Y., Li L., Feng W., Liu F., Zou X., Chen M. (2018). Binocular vision measurement and its application in full-field convex deformation of concrete-filled steel tubular columns. Measurement.

